# WELL-BEING DURING THE COVID-19 PANDEMIC: THE ROLES OF DEMOGRAPHICS, PERSONALITY, AND SOCIAL TIES

**DOI:** 10.1093/geroni/igac059.891

**Published:** 2022-12-20

**Authors:** Lindsay Ryan, Heather Fuller, Aurora Sherman

## Abstract

The COVID-19 pandemic continues to exert widespread impacts on individuals, particularly older adults (Tyrrell & Williams, 2020). This symposium capitalizes on a variety of data sources to advance our understandings of the psychosocial impact of the pandemic on older adults. The first two papers consider the importance of personality characteristics in understanding the effects of social distancing. Fiori et al. highlight the potential for sociability to act as a liability during times of social distancing, finding that sociability exacerbated the effects of social distancing on mental health outcomes in a sample of community-dwelling older adults. Ryan’s paper focuses on the Big Five Personality traits, age, and population density as key characteristics explaining differences in subjective well-being during the pandemic. Next, Van Vleet et al. apply a mixed-methods approach to investigate when older adults expect life to go back to normal, finding that expectations about the future became more positive with the passage of time. The final two papers consider the importance of adults’ home social context during the pandemic. Newton examines relationships between living alone and well-being outcomes among older Canadian women, finding that perceived COVID-19 impact was significant only at T1 and living alone was linked to poorer well-being by T2. Birditt et al. examine how individuals’ and partner’s COVID-19 stress and couples’ racial composition are related to affective experiences measured via ecological momentary assessments, finding that husbands’ stress impacted both partners’ well-being, and that associations differed by race. Sherman will lead a discussion to synthesize these new findings.

